# Proficiency in bariatric surgery may shorten the learning curve for minimally-invasive D2 gastrectomy

**DOI:** 10.1007/s00423-024-03485-8

**Published:** 2024-10-08

**Authors:** Sven Flemming, Lars Kollmann, Anna Widder, Joy Backhaus, Johan Friso Lock, Felix Nickel, Alexander Wierlemann, Armin Wiegering, Christoph-Thomas Germer, Florian Seyfried

**Affiliations:** 1 Department of General, Visceral, Transplantation, Vascular and Pediatric Surgery, Center of Operative Medicine (ZOM), University Hospital of Wuerzburg, Wuerzburg, Germany; 2 Department of Medical Education and Education Research, University of Wuerzburg, Wuerzburg, Germany; 3 Department of General, Visceral, and Thoracic Surgery, University Medical Center Hamburg- Eppendorf, Hamburg, Germany

**Keywords:** Minimally invasive gastrectomy, Bariatric surgery, Robotic surgery, Learning curve

## Abstract

**Introduction:**

Evidence from Asian studies suggests that minimally-invasive gastrectomy achieves equivalent oncological but improved perioperative outcomes compared to open surgery. Oncological gastric resections are less frequent in European countries. Index procedures may play a role for the learning curve of minimally-invasive gastrectomy. The aim of our study was to evaluate if skills acquired in bariatric surgery allow a safe and oncologically adequate implementation of minimally-invasive gastrectomy in a cohort of european patients.

**Methods:**

In this single-center retrospective study, all patients who received primary bariatric surgery between January 2015 and December 2018 and minimally-invasive surgery for gastric cancer treated from June 2019 to January 2023 were evaluated. Primary endpoints were operation time, lymph node yield and lymph node fractions. Secondary endpoints included postoperative complications and oncological outcomes.

**Results:**

Learning curves for two surgeons with 350 bariatric procedures and 44 minimally-invasive gastrectomies were analyzed. For bariatric surgery, the mean operation time decreased from initially 82 ± 27 to 45 ± 21 min and 118 ± 28 to 81 ± 36 min for sleeve gastrectomy (SG) and Roux-en-Y gastric bypass (RYGB), while the complication rate remained within the international benchmark. For laparoscopic gastrectomy (*n* = 30), operation times decreased but then remained stable over time. Operation times for the robotic platform were longer (302 ± 60 vs. 390 ± 48 min; *p* < 0.001) with the learning curve remaining incomplete after 14 procedures. R0 status was achieved in 95.5% of patients; the mean number of lymph nodes retrieved was 37 ± 14 with no differences between the groups. Complete mesogastric excision was more frequently achieved during the later laparoscopic cases whereas it occurred earlier for the robotic group (*p* = 0.004). Perioperative morbidity was comparable to the European benchmark. Textbook outcome was achieved in 54.4% of the cases.

**Conclusion:**

In summary, we could demonstrate a successful skill transfer from bariatric surgery to minimally-invasive laparoscopic oncological gastric surgery enabling safe and oncologically adequate minimally-invasive D2 gastrectomy in a central European patient collective.

## Introduction

Gastric cancer is the fifth most common cancer and remains the third leading cause of cancer-related deaths [1]. In Western societies, the majority of gastric adenocarcinomas are locally advanced and/or nodal positive at diagnosis [2]. Consequently, curative treatment of gastric cancer entails surgical resection with D2 lymphadenectomy and perioperative chemotherapy [1]. The extent of surgical resection depends on tumor localization and includes subtotal, total or transhiatal extended gastrectomy, respectively.

While historically gastrectomy was performed using an open approach, recent evidence suggests that minimally-invasive gastrectomy results in equivalent oncological but improved perioperative outcomes. This includes a shorter length-of-stay (LOS), faster patient recovery and better short-term quality of life [3–5]. However, minimally-invasive D2 gastrectomy is a challenging surgical procedure requiring advanced laparoscopic or robotic skills. Thus, the proficiency level and learning curves of surgeons’ merit particular attention in order to ensure optimal minimally-invasive surgery with equivalent oncological outcomes and improved postoperative recovery compared to open surgery [6, 7].

The concept of a learning curve was first introduced in aircraft manufacturing, and was later transferred and applied to medicine and surgery [8]. Current definitions of learning curves in surgery vary but in summary they describe a learning process with a mandatory number of operations required for each surgeon to independently achieve reasonable outcomes [8]. For minimally-invasive distal and total gastrectomy, it is estimated that 20–100 procedures are needed until the learning curve is complete, which is heavily influenced by institutional and individual surgical cumulative volumes [6, 9, 10].

Most of the available evidence on the learning curves in minimally-invasive oncological gastric surgery derives from studies performed in Asian countries, where the incidence of gastric cancer is high and structured screening programs are established [2]. This leads to higher case volumes with less advanced gastric cancer types, which both affect the learning curve. Notably, the body-mass-index (BMI) of Asian patients is different from that of Western patients, which may further limit the transferability of these results [11, 12] .

While the minimally-invasive approach is still not considered the standard for gastric cancer surgery in Western societies, it has been the standard approach for bariatric surgery for decades [13]. Bariatric surgery is sophisticated and demands advanced skills in minimally-invasive surgery (MIS). A recently published systematic review evaluating learning curves in bariatric surgery demonstrated that mastery is reached after 500 Roux-en-Y-gastric- bypass operations and 100–200 procedures of laparoscopic sleeve gastrectomy, respectively [8]. Bariatric surgeries are considered as Index procedures facilitating the learning curve of other upper gastrointestinal surgeries including oncological gastrectomies [8]. A recent study on hospital experience in bariatric surgery showed an improvement in short-term outcomes after minimally-invasive esophagectomy [14].

The Roux-en-Y-gastric-bypass contains a very similar anatomical rearrangement compared to the reconstruction necessary after oncological D2 gastrectomy. The main aim of our study was to evaluate if the skills acquired in bariatric surgery enable a safe and oncologically adequate minimally-invasive D2 gastrectomy during our first consecutive Western case series. A secondary aim was to determine if proficiency in bariatric surgery could impact on the expected learning curve for minimally-invasive D2 gastrectomy.

## Methods

### Institution

The Department of General, Visceral, Transplantation, Vascular and Pediatric Surgery at the University Hospital of Wuerzburg is certified as a Center of Reference for bariatric and metabolic surgery from the German Society of General- and Visceral Surgery (DGAV) and as Center of Reference for gastric cancer from the German Society of Hospitals (Deutsche Krankenhausgesellschaft). The bariatric center performs more than 150 primary and revisional operative procedures per year on average. All patients referred for bariatric surgery are discussed at a multidisciplinary team meeting including at least an endocrinologist, psychologist/psychiatrist, nutritionist, and bariatric surgeon and are treated according to national guidelines [15]. Patients suffering from gastric cancer are also treated by an interdisciplinary team in line with national and international guidelines for multimodal cancer therapy [16, 17]. On average, 26 oncological gastric resections with curative intent were annually performed at our certified centre during the study period [18]. All gastrectomies had to be performed by one of the three certified surgeons according to the guidelines by the German Cancer Society. Two of the three were also qualified senior bariatric surgeons. There were no specific selection criteria for minimally-invasive surgery but the surgeon’s availability.

### Study population

In this single-center retrospective study, all patients who received bariatric surgery between January 1st, 2015 and December 31st, 2018 (laparoscopic sleeve gastrectomy or Roux-en-Y-gastric-bypass) were included in the analysis. Patient characteristics and operative data were also collected from patient records. Patients with re-do and conversion operations were excluded.

All consecutive patients with subtotal, total and transhiatal extended minimally-invasive surgery for gastric cancer without distant metastases treated from June 1, 2019 to January 31, 2023 at the Department of General, Visceral, Transplantation, Vascular and Pediatric Surgery at the University Hospital of Wuerzburg were evaluated. Sociodemographic, clinicopathological and operative data including postoperative outcome were collected for each patient from patient records. An institutional review board approval was not applicable for our study.

### Surgeons

All minimally-invasive D2 gastrectomies were performed by two surgeons who had previously performed more than 100 laparoscopic sleeve gastrectomy and 150 Roux-en-Y-gastric-bypass surgeries within the last 5 years. Both surgeons performed more than 20 open D2 gastrectomies before starting minimally-invasive oncological gastric resections. Before starting with robotic D2 gastrectomies the two surgeons gained experience of > 50 robotic procedures mainly consisting of functional upper gastrointestinal and RYGB surgery.

### Operative technique

The decision to perform total, subtotal or transhiatal extended gastrectomy was made according to the tumor location, intraoperative frozen section analyses and technical feasibility. D2 lymphadenectomy with omentectomy and simultaneous cholecystectomy was performed on all oncological patients. The goal was to achieve complete mesogastric excision [19]. Reconstruction was done intracorporally in a Roux-en-Y fashion. End-to-side esophago-jejunostomy was performed if gastrectomy (including transhiatally extended) was necessary, a side-to side gastrojejunostomy if subtotal gastrectomy was possible. The retrocolic alimentary and biliopancreatic limb were anastomosed in a side-to-side fashion. All anastomoses were stapled with EndoGIA^®^ ultra 60 mm purple linear magazines (Medtronic, USA). Esophago-jejunostomies were stapled with 25 mm circular staplers (Ethicon, Hamburg, Germany or OrVil™, Medtronic, USA). Mesenterial defects were closed using non-absorbable sutures. The Da Vinci Xi Surgical System (Intuitive Surgical, Sunnyvale, CA, USA) was used to perform the robotic gastrectomies.

### Outcome

The primary endpoint was defined as the operation time (including docking time if the operation was performed robotically). Secondary endpoints were lymph node yield (total number of lymph nodes), resected fraction of specimen postoperative complications according to Clavien-Dindo grade >/=IIIa, LOS, hospital readmission, MTL30 [20] (mortality, transfer, length of stay), and oncological outcomes comprised TNM classification and R-status and textbook outcome [22].

### Statistical analysis

Descriptive data is presented as median with standard deviation or total numbers with percentage. Differences in patient characteristics were assessed by Chi-Square test, Fisher`s exact test or ANOVA test according to data scale and distribution. A *p*-value of < 0.05 was considered statistically significant. Statistical analysis was performed using SPSS statistics (Version 29, IBM, Armonk, NY, USA). Figures were made by SPSS statistics (Version 29, IBM, Armonk, NY, USA, and GraphPad Prism (Prism 9 for macOS, Version 9.5.0), respectively.

### CUSUM analysis

To inspect performance of technique “robotic” compared to technique “laparoscopy”, each number of operative time, lymph nodes fraction and textbook outcome was subtracted from the group median prior to calculation of the cumulative sum (CUSUM). Consequently, the value zero represents the predetermined reference level, also referred to as the target level. 95% confidence intervals (CI) based either on the generalized linear model or local CUSUM nal regression are plotted as gray shading. Once the CUSUM curve leaves the CI, significant deviations from the median occur.

## Results

### Bariatric patient characteristics

In this single-center study, 350 bariatric patients receiving either primary laparoscopic sleeve gastrectomy (*n* = 131) or laparoscopic Roux-en-Y-gastric-bypass (*n* = 219) were included to evaluate the learning curve of minimally-invasive bariatric surgery between January 1, 2015 and December the 31, of 2018. This represents the only primary bariatric surgery cases performed by the two surgeons who later performed minimally-invasive oncologic D2 gastrectomies.

Patient characteristics of both groups are presented in Table 1. Patients in the sleeve gastrectomy group were older (47 vs. 43 years; *p* = 0.014), had a higher body-mass-index (BMI) (54.8 vs. 47.1; *p* < 0.001), higher rates of ASA classification ≥ III (72.5% vs. 51.1%; *p* < 0.001), of type 2 diabetes (59.5% vs. 47.5; *p* = 0.014) of obstructive sleep apnea syndrome (OSAS) (50.4% vs. 34.7%; *p* < 0.001), and of liver steatosis ( 26.7% vs. 18.7%; *p* = 0.044), as well as a higher Edmonton Obesity Scoring System (EOSS) score ≥ 3 (28.2% vs. 17.8%; *p* = 0.013).

**Table 1:** Baseline characteristics of patients who underwent bariatric surgery. ASA: American Society of Anesthesiology; CCI: Charlson comorbidity index; EOSS: Edmonton obesity scoring system; OSAS: obstructive sleep apnea syndrome

	Sleeve Gastrectomy	Roux-en-Y gastric bypass	*p*-value
Number (n)	131	219	-
Age (mean, range; years)	47 (22–67)	43 (19–67)	**0.014**
Sex (n, %)			
Female Male	85 (65) 46 (35)	164 (74.9) 55 (25.1)	0.051
BMI, mean (SD), kg/m^2^	54.8 (35–80)	47.1 (34–76)	**< 0.001**
ASA classification ≥ III (n, %)	95 (72.5)	112 (51.1)	**< 0.001**
CCI (mean, range)	2 (0–8)	2 (0–8)	1
Type 2 Diabetes mellitus (n, %)	78 (59.5)	104 (47.5)	**0.014**
OSAS (n, %)	66 (50.4)	76 (34.7)	**< 0.001**
Liver steatosis (n, %)	35 (26.7)	41 (18.7)	**0.044**
Chronic kidney failure (n, %)	9 (6.9)	11 (5.0)	0.244
EOSS Score ≥ 3 (n, %)	37 (28.2)	39 (17.8)	**0.013**

### Gastric cancer patient characteristics

In total, 44 patients received minimally-invasive D2 subtotal, total or transhiatal extended gastrectomy. Of those, 30 patients were identified with laparoscopic D2 gastric cancer resection comprising 6 patients with total gastrectomy, 15 patients with subtotal gastrectomy, and 9 patients with transhiatal extended gastrectomy. For further analysis, this patient group was divided according to patients operated in the first year (*n* = 15) and patients operated in the second year (*n* = 15) of the observational period. As presented in Table 2, both groups did not show any significant differences regarding age, gender, BMI, and tumor localization. There were only slight differences with a higher Charlson Comorbidity Index (CCI) in the laparoscopic group since patients from the first year were more often diagnosed with type 2 diabetes mellitus (40% vs. 6.7%; *p* = 0.005). Furthermore, no differences in neoadjuvant therapy and extension of surgical resection (subtotal vs. total vs. transhiatal extended gastrectomy) were detected. During the observational period, 14 patients underwent robotic D2 gastrectomy who showed similar baseline characteristics including tumor location, neoadjuvant therapy and dimension of resection compared to patients operated laparoscopically as shown in Table 2.

**Table 2:** Baseline characteristics of patients who underwent laparoscopic and robotic gastrectomy. Laparoscopic gastrectomy patients are divided in group 1st year and group 2nd year. Cardiovascular risk factors include arterial hypertension, coronary heart disease, and peripheral arteriosclerosis. Pulmonary risk factors encompass asthma and chronic obstructive pulmonary disease (COPD). ASA: American Society of Anesthesiology, CCI: Charlson comorbidity index; SD: standard deviation; * *p*-value between 1st and 2nd year group

	All	Laparoscopic gastrectomy 1st year	Laparoscopic gastrectomy 2nd year	Robotic gastrectomy	*p*-value*
Number	44	15	15	14	
Age (median ± SD; years)	66.41 (± 11.6)	70.8 (± 11.8)	67.0 (± 9.3)	61.1 (± 12.5)	0.073
Sex (n, %)					
Female Male	11 (25.0%) 33 (75.0%)	1 (6.7%) 14 (93.3%)	6 (40%) 9 (60%)	4 (28.6%) 10 (71.4%)	0.101
Body Mass Index (median ± SD; kg/m^2^)	28.7 (± 5.7)	27.6 (± 3.2)	30.4 (± 7.4)	28.1 (± 5.7)	0.351
Type 2 Diabetes mellitus (n, %)	7 (15.9%)	6 (40%)	1 (6.7%)	0 (0%)	**0.006**
Cardiovascular risk factors (n, %)	25 (56.8%)	10 (66.7%)	9 (60%)	6 (42.9%)	0.413
Pulmonary risk factor (n, %)					
COPD (n, %) Asthma (n, %)	5 (11.4%) 2 (4.5%)	1 (6.7%) 0 (0%)	3 (20%) 1 (6.7%)	1 (7.1%) 1 (7.1%)	0.575
Liver disease (n, %)					
Hepatic steatosis (n, %) Cirrhosis (n, %)	3 (6.8%) 1 (2.3%)	2 (13.3%) 1 (6.7%)	1 (6.7%) 0 (0%)	0 (0%) 0 (0%)	0.385
Charlson Comorbidity Index (median ± SD)	4.78 (± 1.7)	5.73 (± 1.7)	4.60 (± 1.2)	4.00 (± 1.8)	**0.017**
ECOG (median ± SD)	0.75 (± 0.5)	0.87 (± 0.4)	0.73 (± 0.6)	0.64 (± 0.6)	0.534
ASA score (median ± SD)	2.61 (± 0.54)	2.8 (± 0.41)	2.53 (± 0.52)	2.50 (± 0.65)	0.256
Smoking (n, %)	12 (27.3%)	3 (20%)	4 (26.7%)	5 (35.7%)	0.636
Tumor localization					
Cardias Corpus Antrum	14 (31.8%) 18 (40.9%) 12 (27.3%)	5 (33.3%) 6 (40%) 4 (26.7%)	4 (26.7%) 6 (40%) 5 (33.3%)	5 (35.7%) 6 (42.9%) 3 (21.4%)	0.963
Neoadjuvant therapy					
None Yes	15 (34.1%) 29 (65.9%)	5 (33.3%) 10 (66.7%)	7 (46.7%) 8 (53.3%)	3 (21.4%) 11 (78.6%)	0.357
History of previous abdominal open surgery (n, %)					
None Yes	42 (95.5%) 2 (4.5%)	15 (100%) 0 (0%)	14 (93.3%) 1 (6.7%)	13 (92.9%) 1 (7.1%)	0.581
Surgical technique (n, %)					
Subtotal gastrectomy Total gastrectomy Transhiatal extended	23 (52.3%) 7 (15.9%) 14 (31.8%)	7 (46.7%) 3 (20%) 5 (33.3%)	8 (53.3%) 3 (20%) 4 (26.7%)	8 (57.1%) 1 (7.1%) 5 (35.7%)	0.853

### Postoperative outcomes of bariatric surgery

**Fig. 1:**
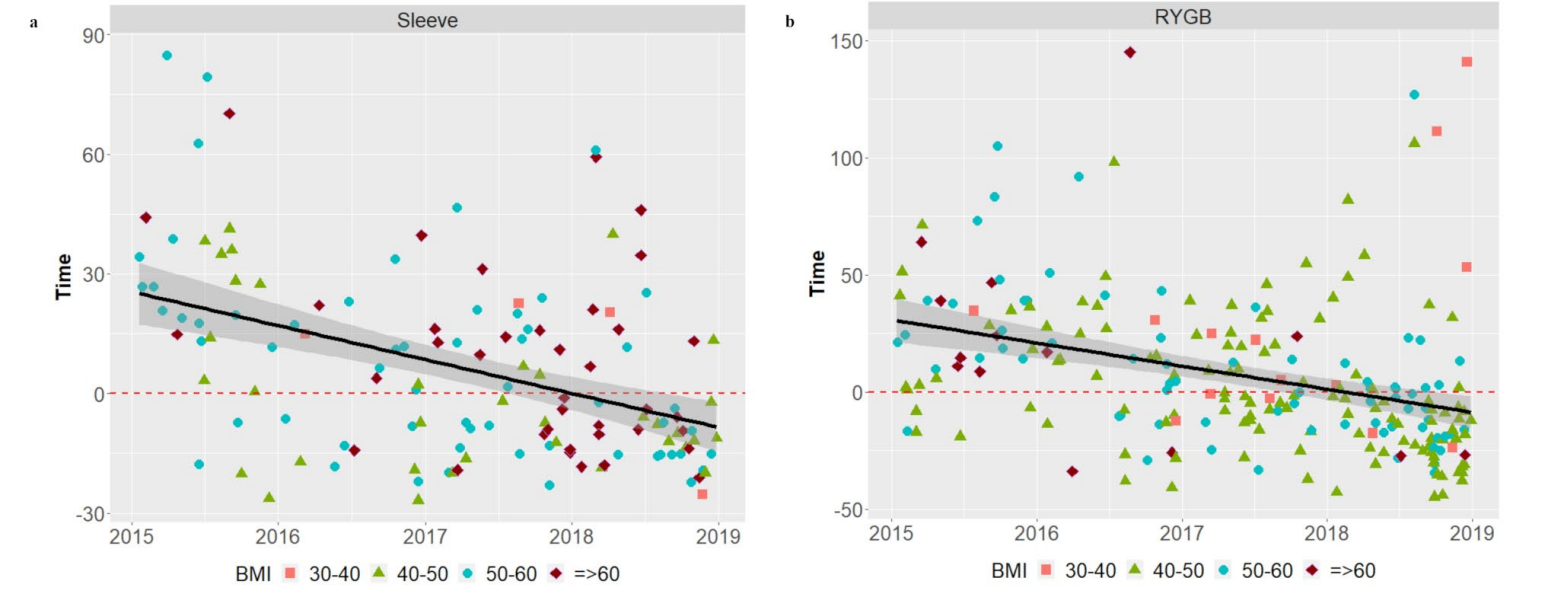
(**a**) Learning curve in respect of operation time in laparoscopic sleeve gastrectomy. BMI (Body-mass-index in kg/m²). Y-axis: time in minutes. (**b**) Learning curve in respect of operation time in laparoscopic Roux-en-Y gastric bypass. BMI (Body-mass-index in kg/m²). RYGB (Roux-en-Y gastric bypass). Y-axis: time in minutes

The mean operation duration significantly decreased over time for both procedures from initially 82 ± 27 to 45 ± 21 min and 118 ± 28 to 81 ± 36 min for sleeve gastrectomy (sleeve) and Roux-en-Y gastric bypass (RYGB), respectively, in 2015 (Table 3). This effect could be determined for both learning curves of laparoscopic sleeve and RYGB as shown in Fig. 1a and b. The decrease of operative time was not accompanied with increased morbidity as indicated by postoperative complications (Table 3). Of note, there were no differences among the two different surgeons regarding patient characteristics, perioperative outcome and learning curve.

**Table 3:** Outcomes of bariatric surgery. SG: sleeve gastrectomy; RYGB: Roux-en-Y gastric bypass; SD: standard deviation

	2015	2016	2017	2018	Overall
Surgical technique (n, %) SG RYGB	68 29 (42.6) 39 (57.4)	64 22 (34.4) 42 (65.6)	88 38 (43.2) 50 (56.8)	130 42 (32.3) 88 (67.7)	350 131 (37.4) 219 (62.6)
Operative time (median ± SD; min)					
SG RYGB	82 ± 27 118 ± 28	56 ± 18 107 ± 37	53 ± 17 94 ± 21	45 ± 21 81 ± 36	59 ± 20 93 ± 31
Length of hospital stay (median ± SD, days)					
SG RYGB	5 ± 0.8 6 ± 3.5	5 ± 0.9 5 ± 1.3	5 ± 7.8 5 ± 1.1	5 ± 5.1 5 ± 2.9	5 ± 2.7 5 ± 2.1
Postoperative complications (Clavien-Dindo grade > IIIa; n, %)					
SG RYGB	1 (3.4) 1 (2.6)	0 (0.0) 1 (2.4)	2 (5.2) 1 (2.9)	4 (9.5) 6 (6.8)	7 (5.3) 9 (4.1)
Anastomotic leakage					
SG RYGB	0 (0.0) 1 (2.6)	0 (0.0) 0 (0.0)	0 (0.0) 1 (2.0)	0 (0.0) 1 (1.1)	0 (0.0) 3 (1.3)

### Postoperative outcome of minimally-invasive gastrectomy

**Fig. 2:**
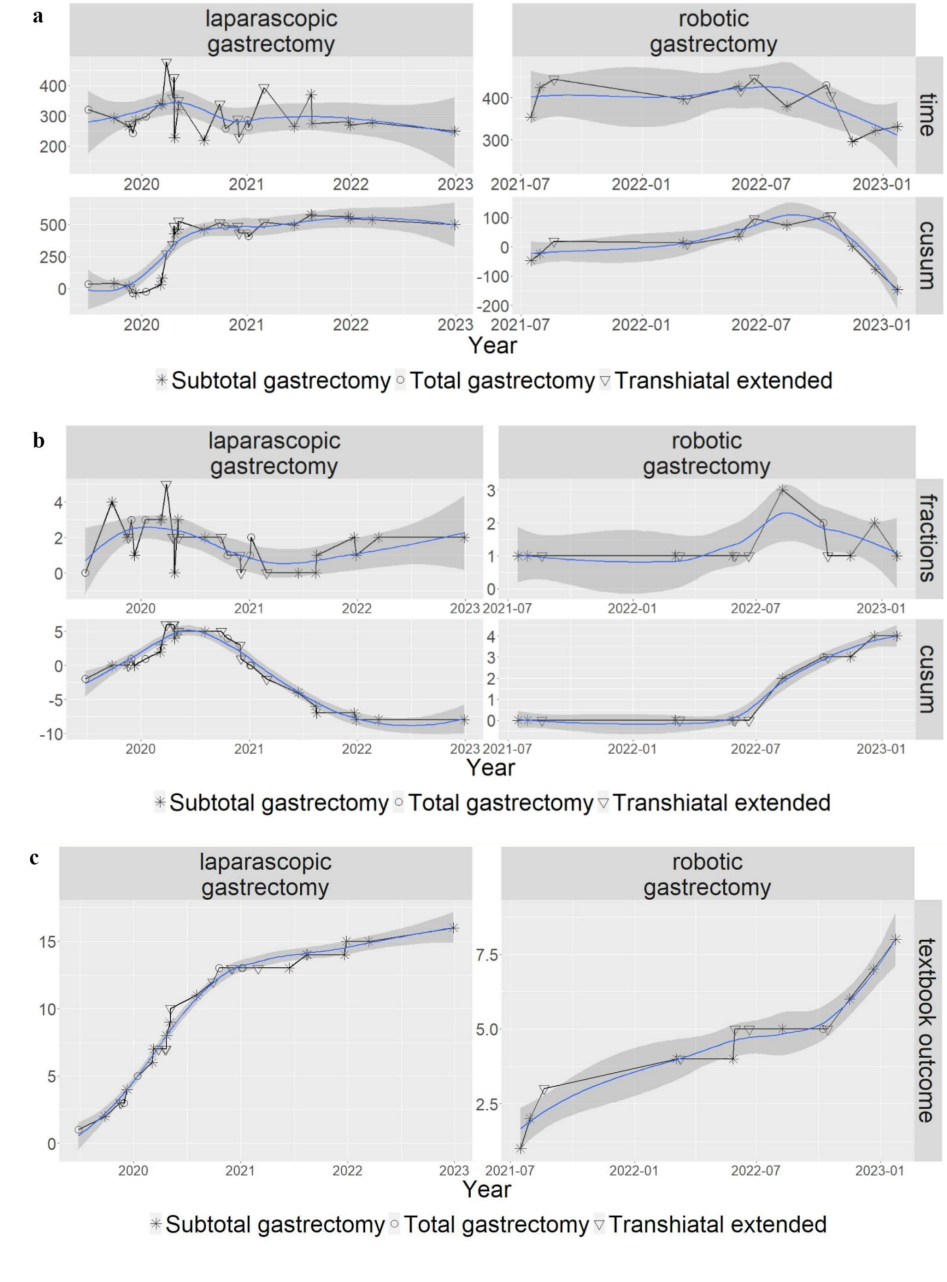
(**a**) Operative Time (upper) and learning curve (CUSUM curve, lower) of laparoscopic and robotic gastrectomy in respect of operation time. Circle: total gastrectomy; triangle: trans-hiatal gastrectomy; star: subtotal gastrectomy. (**b**) Total number of specimen fractions (triangle: trans-hiatal gastrectomy; circle: total gastrectomy; square: subtotal gastrectomy), and associated learning curve (CUSUM curve; circle: total gastrectomy: triangle: trans-hiatal gastrectomy; star: subtotal gastrectomy). (**c**) Textbook outcome of oncologic gastric resections. (CUSUM curve; circle: total gastrectomy: triangle: trans-hiatal gastrectomy; star: subtotal gastrectomy)

Analyzing mean operation time showed no significant differences between 1st and 2nd year of laparoscopic gastrectomy (321 ± 66 vs. 284 ± 48 min; *p* = 0.205), but significantly longer operation time in patients operated robotically compared to the laparoscopic approach (302 ± 60 vs. 390 ± 48 min; *p* < 0.001). Of note, there were no differences among the two different surgeons regarding patient characteristics, perioperative outcomes and the learning curve. However, the learning curve revealed that operation times slightly decreased, then became variable but remained stable. The learning curve for the robotic platform showed the same pattern but further decreased during the later course indicating that the learning curve had not been completed (Fig. 2).

Postoperative complications are summarized in Table 4. Overall, two patients required re-operation of whom one patient had a postoperative anastomotic bleeding and one patient suffered from duodenal stump leakage with consecutive acute hemorrhage. Five patients suffered from anastomotic leakage of which four received transhiatal extended resection and one patient a gastrectomy (Table 4).

**Table 4:** Postoperative outcome after minimal-invasive gastrectomy MTL 30, mortality, transfer, length-of-stay > 30 days; SD: standard deviation, * *p*-value between 1st and 2nd year group

	All	Laparoscopic gastrectomy 1st year	Laparoscopic gastrectomy 2nd year	Robotic gastrectomy	*p*-value*
Operation time (median ± SD; min)	331 (± 69.5)	321 (± 66.3)	284 (± 48.2)	391 (± 47.6)	**< 0.001**
Length of hospital stay (median ± SD, day)	15.9 (± 9.2)	14.9 (± 7.6)	17.7 (± 9.6)	15.3 (± 10.5)	0.677
Postoperative complications (Clavien-Dindo grade IIIb-IVb; n, %)					
Anastomotic leakage Leakage associated sepsis Leakage associated mortality Anastomotic bleeding Duodenal leakage Re-operation	5 (11.4%) 0 (0%) 0 (0%) 1 (2.3%) 1 (2.3%) 2 (4.5%)	1 (6.7%) 0 (0%) 0 (0%) 1 (6.7%) 0 (0%) 1 (6.7%)	2 (13.3%) 0 (0%) 0 (0%) 0 (0%) 0 (0%) 0 (0%)	2 (14.3%) 0 (0%) 0 (0%) 0 (0%) 1 (7.1%) 1 (7.1%)	0.777 1 1 0.372 0.334 0.581
Mortality (within 30 days; n, %)	1 (2.3%)	0 (0%)	0 (0%)	1 (7.1%)	0.320
MTL30 (n, %)	7 (15.9%)	1 (6.7%)	3 (20%)	3 (21.4%)	0.481
Hospital readmission (within 30 days) (n, %)	2 (4.5%)	1 (6.7%)	0 (0%)	1 (7.1%)	0.581
Textbook outcome (n,%)	24 (54.5)	10 (66.6%)	6 (40%)	8 (57.1%)	0.298

However, all cases were successfully treated by endoluminal vacuum therapy (EVT) without further surgical revision or septic complications (Table 5).

**Table 5:** Postoperative anastomotic leakage therapy. SD: standard deviation. D: days

	Subtotal gastrectomy & Total gastrectomy	Transhiatal extended	*P* value
Anastomotic leakage	1/30 (3.3%)	4/14 (28.6%)	**0.022**
Endoscopic vacuum treatment (mean ± SD; d)	7 (± 0.0)	14.25 (± 6.9)	0.419
Textbook outcome	20/30 (66.6%)	4/14 (28.6%)	**< 0.001**

The length of hospital stay was 15.6 ± 9.2 days overall (14.9 ± 7.6 days in laparoscopic 1st year vs. 17.7 ± 9.6 days in 2nd year vs. 15.3 ± 10.5 days in the robotic group; *p* = 0.677). The readmission rate was 4.5% with one patient in the robotic and one in the 1st laparoscopic group (*p* = 0.581). One patient died within 30 days after surgery due to acute pulmonary-artery embolism. During the implementation process, complications occurred independently of the number of cases being performed. Quality of oncological resection for minimally-invasive D2 gastrectomy.

Histopathological examination of specimens revealed no differences in T- and N-categories in laparoscopic and robotic groups. Frozen sections of resection margins showed R0 resection in all cases. In two patients with laparoscopic transhiatal extended gastrectomy, the final histopathological examination revealed R1 resection status, which resulted in definite R0 status in 95.5% of patients. The mean number of lymph nodes retrieved was 37 ± 14 with no differences between the three groups. Complete mesogatric excision was more frequently achieved during the second year and in the robotic group (1.1 fractions) compared to the first year (2.3 fractions, *p* = 0.004). All results are shown in Table 6.

**Table 6:** Oncological outcomes after minimal-invasive gastrectomy. SD: standard deviation, * *p*-value between 1st and 2nd year group

	All	Laparoscopic gastrectomy 1st year	Laparoscopic gastrectomy 2nd year	Robotic gastrectomy	*p*-value*
T category (n, %)					0.205
T0 T1 T2 T3 T4	3 (6.8%) 11 (25.0%) 7 (15.9%) 17 (38.6%) 6 (13.6%)	1 (6.7%) 7 (46.7%) 1 (6.7%) 4 (26.7%) 2 (13.3%)	1 (6.7%) 1 (6.7%) 3 (20%) 6 (40%) 4 (26.7%)	1 (7.1%) 3 (21.4%) 3 (21.4%) 7 (50.0%) 0 (0.0%)	
Nodal status (n, %)					0.396
N0 N1 N2 N3	22 (50.0%) 12 (27.3%) 6 (13.6%) 4 (9.1%)	9 (60%) 2 (13.3%) 2 (13.3%) 2 (13.3%)	8 (53.3%) 3 (20%) 3 (20%) 1 (6.7%)	5 (35.7%) 7 (50.0%) 1 (7.1%) 1 (7.1%)	
Number of lymph nodes (median ± SD)	36.7 (± 14.1)	39.7 (± 13.9)	37.6 (± 13.7)	32.6 (± 14.8)	0.386
Number of lymph node fractions (median ± SD)	1.6 (± 1.2)	2.3 (± 1.3)	1.1 (± 0.8)	1.1 (± 0.9)	**0.004**
Surgical margin (n, %)					0.132
R0 R1	42 (95.5%) 2 (4.5%)	15 (100%) 0 (0%)	13 (86.7%) 2 (13.3%)	14 (100%) 0 (0%)	

### Textbook outcome in MIS D2 gastrectomy

Textbook outcome as defined by Busweiler et al. [22] was reached in 24/44 patients 54.4%, with no differences between the laparoscopic and robotic group. Of note, textbook outcome was reached in 20/30 66.6% in patients after total and subtotal gastrectomy but only in 4/14 (28.6%) in patients with transhiatal extended resections. CUSUM analysis showed an inclination after 16 cases in laparoscopic but none after 14 robotic MIS D2 gastrectomies (Fig. 2c).

## Discussion

Performing laparoscopic RYGB surgery quickly builds up experience for necessary reconstructive and anastomotic techniques to successfully perform MIS D2 gastrectomy. Our learning curve for Roux-en-Y gastric bypass is not different to what has been published before [8]. We then observed a steep learning curve for MIS D2 gastric resections which is in contrast to what has been published before with learning curves ranging from 25 to 100 cases [6].

Thus, the necessary learning curve for RYGB could have contained the anticipated learning curve for MIS D2 gastrectomy. Therefore, we hypothesized - but of course did not prove - that the RYGB could serve as a valuable index procedure before learning MIS D2 gastrectomy.

As most high-quality studies comparing minimally-invasive versus open gastric cancer surgery are derived from Asia. These studies have shown that the oncological and perioperative outcomes were similar while patient recovery was enhanced when the minimally-invasive approach was used [4, 5]. However, these studies also revealed that a certain level of surgical experience in minimally-invasive surgery is mandatory to achieve optimal outcomes [6]. It has remained questionable if these results can be easily transferred to Western European countries [21], which led to the initiation of a European prospective randomized multicenter trial comparing the minimally-invasive versus the open approach for oncological D2 gastrectomy [11]. The requirements for the participating surgeons include the successful performance of at least 20 procedures with the open, laparoscopic and robotic platforms. As we aimed to participate in this trial, we took this as an opportunity to analyze our learning curve and to review our own results during the implementation of minimally-invasive gastric cancer surgery and to compare our learning curve with those published in the literature [22, 23, 24, 25]. We assumed that our previous laparoscopic skills with a mastery level for bariatric surgery could enable us to perform safe and oncologically adequate, minimally-invasive D2 gastrectomies early on as suggested by the concept of index procedures. Further, we reasoned that surgeons with sufficient experience in laparoscopic gastrectomy can rapidly overcome the learning curve for robotic gastrectomy [6]. Our idea was supported by a recent study which analyzed the impact of experience in bariatric surgery on minimally-invasive esophagectomy, that showed an positive effect on the short-term outcomes, if competence in bariatric surgery was given [14].

Within our bariatric cohort, we observed the expected learning curve with a consistent decrease in operative times. Additionally, complications rates constantly ranged within the international benchmark despite our patient collective being considerably older, more obese and having more comorbidities [26]. The primary outcome of our study was the operative time of minimally-invasive oncological gastrectomy. Operative times decreased during the first procedures of laparoscopic gastrectomy reflecting the learning curve. However, after the first five surgeries a large variation of operation times remained. From our point of view, this may more likely reflect the technical demands of each individual procedure (e.g. transhiatal extended resection with positive resection margin and further subsequent resection) rather than representing the surgeon’s learning curve. This variation is even more pronounced during the second year, which can be explained by the fact that technically more demanding procedures (e.g. in morbidly obese patients) were also performed laparoscopically. As a result, operations times remained longer compared to Asian studies but were faster compared to a Western patient collective with high rates of locally advanced and nodal positive tumors and an obesity diagnosis [21].

As expected, we observed longer operation times when the robotic platform was used, which is in line with previous studies [27]. This can be well explained by setup issues such as the docking process and the longer duration of changing instruments [28]. More importantly, our learning curve is still incomplete. Multiple studies demonstrated that operation times and complications were higher during the first 35 cases to complete the learning curve [29]. Another study by Kim et al. described a learning curve of up to 90 cases needed to reach a mastery level for robotic gastrectomy. In this study, a decrease in complication rate after 25 cases was achieved but there was an increase between cases No.66–88. The lowest complication rates occurred after 89 cases when the highest level of competence was finally achieved [30].

Importantly, during the implementation of laparoscopic and robotic D2 gastrectomies, we consistently found an appropriate oncological quality determined by two surrogate parameters: number of lymph nodes retrieved and the R0 status. Of note, the number of lymph nodes was significantly higher than the required average amount [16, 31]. From our point of view, the achievement of a complete en-bloc D2 resection with complete mesogastric excision could serve as a more appropriate factor to reflect the surgeon’s experience [19]. During the implementation of minimally-invasive gastric cancer surgery, this was achieved more often after the first 15 procedures. Several lymph node fractions were also removed separately as part of the D2 lymphadenectomy.

Using the robotic platform, we were able to achieve complete en-bloc D2 resection with complete mesogastric excision early on with no further improvements over time, which may reflect technical advantages of this platform due to the wristed instruments.

In our first minimally-invasive D2 gastrectomy cohort, perioperative morbidity was comparable to the European benchmark, even though our patients were older and had significantly more severe comorbidity [31].

Among the postoperative complications requiring interventions, five patients diagnosed with a leakage of the oral anastomosis (11.4%) were identified. All of these healed under endoscopic vacuum therapy (EVT) without septic complications. At this point, it should be mentioned that our threshold to perform endoscopic investigations after upper gastrointestinal surgery is considerably low (triggered by increased or non-decreasing infection parameters) and that EVT is liberally started if an anastomotic leak cannot be ruled out during endoscopy [32]. We also observed a considerably high leak rate after transhiatal, extended D2 gastrectomy compared to subtotal or total gastrectomies. For transhiatal resection, an OrVil^™^ device was used as previously described [33]. Three out of four anastomotic leakages in transhiatal extended gastrectomies occurred while using the OrVil^™^ device. Hereafter, we adjusted our anastomotic stapling technique to endoscopic transgastric placement of the 25 mm circular stapler anvil to correctly place the anvil for connection. Afterwards the leakage rate in transhiatal extended gastrectomies improved. Despite previously published case series showing good results using OrVil^™^ device for total gastrectomy [33, 34], data on transhiatal extended resections are limited. Our data suggests that the esophagojejunostomy after transhiatal extended gastrectomy may contain some technical challenges still requiring additional research.

The LOS was considerably longer than previously described but shorter compared to other German studies published before [12, 21, 35]. Admittedly, a standardized protocol ‘ready to discharge’ was not established during the observation period.

Textbook outcome was reached in 54.4% overall and in 66.6% for patients with subtotal or total D2 gastrectomy with no differences among laparoscopic and robotic surgery, which is in line with previous reports of large cohorts and register studies [22] Thus, previous favourable outcomes of MIS D2 gastrectomy were reproduced within our learning curve in a western cohort with patients being diagnosed with advanced tumour stages and considerable comorbidities. The learning curve was vastly completed after 16 in laparoscopic but ongoing after 14 robotic resections (shown in Fig. 2). This observation may support the defined requirements on surgical expertise to participate in a new prospective randomized controlled trail comparing open with MIS D2 gastrectomy [11]. We have to admit that patients with transhiatal extended resections had far inferior outcome (textbook outcome in 28.6%). However, data regarding ‘textbook outcome’ on MIS transhiatal extended D2 gastric resections is scare [36]. Therefore, efforts should be made to gain consistent data on this subject but also to further improve technical aspects for this type of surgery in future studies.

Furthermore, data of LOS are difficult to compare since most patients underwent surgical resection during the COVID-19 pandemic resulting in limited postoperative rehabilitation and outpatient care options, which may have resulted in extended hospital stay after surgery. This study contains several limitations. Firstly, it is a single-center retrospective study. We have to admit that our study does not contain a surgical control group (surgeons with no experience in bariatric surgery) and this is certainly the main limitation. However, as the surgeon’s expertise can be considered as the summary of all her/his experience over the entire course of her/his career [8], it would be difficult to create a matched surgical control that only lacks experience in bariatric surgery. At least this would be difficult in our hospital setting due to the organization of surgical training. It could be attempted to analyse this in a multicentric setting comparing hospitals that do not have a bariatric surgery program and thus train their surgeons in minimally invasive gastrectomy without prior experience in bariatric surgery.

Our observation, that the RYGB could serve as a valuable index procedure before learning MIS D2 gastrectomy, should be considered rather hypothesis creating than being a robust meaningful conclusion. We believe that our results are, nevertheless, worthy being communicated despite the hypothesis needs to be proven in future multicentre studies.

This study could also be noticed as an appeal to use available expertise from bariatric surgeons in complementary sections in times of progressing subspecialisation. In this context, hospitals with experience in bariatric surgery have been shown to have better short-term outcomes after minimally invasive esophagectomy [14].

## Conclusion

We were able to show a successful skill transfer from bariatric surgery to minimally-invasive laparoscopic oncological gastric surgery in a central European patient collective. We assume that our learning curve for bariatric surgery and in particular the Roux-en-Y gastric bypass procedure further shortens the learning curve for the laparoscopic D2 gastric resections. This may underline the educational importance of bariatric surgery for standardized laparoscopic skill training, which then could be transferred to more complex minimal-invasive oncological gastric interventions. However, further investigations in prospective comparative studies are necessary to confirm our single-center observations.

## Data Availability

No datasets were generated or analysed during the current study.
